# Non‐stimulated regions in early visual cortex encode the contents of conscious visual perception

**DOI:** 10.1002/hbm.25731

**Published:** 2021-12-03

**Authors:** Bianca M. van Kemenade, Gregor Wilbertz, Annalena Müller, Philipp Sterzer

**Affiliations:** ^1^ Centre for Cognitive Neuroimaging Institute of Neuroscience and Psychology, University of Glasgow Glasgow UK; ^2^ Department of Psychiatry and Psychotherapy Philipps‐University Marburg Marburg Germany; ^3^ Department of Psychology Freie Universität Berlin Berlin Germany; ^4^ Department of Experimental and Biological Psychology University of Potsdam Potsdam Germany; ^5^ Department of Psychiatry and Psychotherapy Charité Campus Mitte Berlin Germany

**Keywords:** bistable perception, feedback processing, fMRI, MVPA, predictive coding

## Abstract

Predictions shape our perception. The theory of predictive processing poses that our brains make sense of incoming sensory input by generating predictions, which are sent back from higher to lower levels of the processing hierarchy. These predictions are based on our internal model of the world and enable inferences about the hidden causes of the sensory input data. It has been proposed that conscious perception corresponds to the currently most probable internal model of the world. Accordingly, predictions influencing conscious perception should be fed back from higher to lower levels of the processing hierarchy. Here, we used functional magnetic resonance imaging and multivoxel pattern analysis to show that non‐stimulated regions of early visual areas contain information about the conscious perception of an ambiguous visual stimulus. These results indicate that early sensory cortices in the human brain receive predictive feedback signals that reflect the current contents of conscious perception.

## Graphical abstract

The theory of predictive processing poses that our brains make sense of incoming sensory input by generating predictions, which are sent back from higher to lower levels of the processing hierarchy. Here, we used functional magnetic resonance imaging and multivoxel pattern analysis to show that non‐stimulated regions of early visual areas contain information about the conscious perception of an ambiguous visual plaid stimulus (pattern vs component perception). These results indicate that early sensory cortices in the human brain receive predictive feedback signals that reflect the current contents of conscious perception.

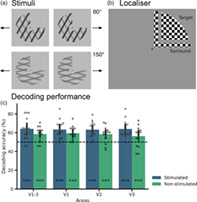

## 1 INTRODUCTION

Predictions play an important role in perception (de Lange, Heilbron, & Kok, 2018). According to the theory of predictive processing, our brains use an internal model of the world to make predictions that are fed back from higher to lower levels of the processing hierarchy, thereby enabling inferences about the hidden causes of the sensory input data (Friston, 2005; Rao & Ballard, 1999). This framework might provide the key to a neuroscientific account of conscious perceptual experiences, one of the greatest challenges for theories of human brain function. Within the framework of predictive processing, it has been proposed that conscious perception corresponds to the currently most probable internal model of the world, that is, the model that makes the best predictions about the incoming sensory data (Hohwy, Roepstorff, & Friston, 2008). From this conceptualization of conscious perception as reflecting a predictive model, it follows that predictions generated by this model should be fed back from higher to lower levels of the processing hierarchy.

In the current study, we investigated whether predictive feedback signals that reflect the current contents of conscious perception can be observed in non‐stimulated regions of human early visual cortex. Non‐stimulated visual regions do not receive any bottom‐up stimulation; therefore, any information in these regions must come from higher visual areas through feedback connections. This approach has successfully been used in several previous studies, showing for example that feedback signals contain information not only about which visual scene is presented (Smith & Muckli, 2010), but also about the spatial frequency of the scene (Revina, Petro, & Muckli, 2017). High‐field fMRI studies have confirmed that decoded information in non‐stimulated visual areas is due to feedback mechanisms, as this information was present in superficial cortical layers, where feedback signals arrive, and not the middle cortical layers, which process feedforward input (Muckli et al., 2015). Measuring neural activity in regions of retinotopic visual cortex that do not receive feedforward input thus provides an elegant way to isolate effects of predictive feedback signalling in the human brain.

Here, we used this method to probe whether the actual contents of conscious visual perception, too, would be reflected by neural signals in non‐stimulated regions of early visual cortex. We used an ambiguous motion stimulus that gives rise to bistable perception (i.e., spontaneous alternations between two perceptual states) and that was partially occluded. The stimulus consisted of two superimposed gratings moving in different directions, which could be interpreted either as two gratings moving in their respective directions (component perception) or as a plaid moving in the average direction of the two gratings (pattern perception). Decoding the two perceived visual interpretations of the constant ambiguous stimulus, rather than two distinct stimuli, from non‐stimulated visual regions would thus enable us to identify the presence of feedback signals reflecting the current conscious percept. Area hMT+/V5 has been reported to be differentially activated during component versus pattern motion (Castelo‐Branco et al., 2002; Grassi, Zaretskaya, & Bartels, 2018) and is therefore a likely candidate for the origin of these feedback signals. Therefore, we also decoded from area hMT+/V5, and performed univariate ROI analyses both on stimulated and non‐stimulated regions in early visual cortex and on area hMT+/V5 to better understand the neural processes underlying bistable plaid perception.

## 2 METHODS

### 2.1 Subjects

Sixteen participants took part in the study. Data from one participant had to be excluded, because this participant reported only one percept in certain conditions, so that the other percept of the respective condition could not be modelled (see Section 2.6). This resulted in a final sample of 15 participants (age 18–33, M = 23.5 years, *SD* = 4.22, 5 male). None of the participants reported current or previous neurological or psychiatric disorders. All had normal or corrected‐to‐normal vision and were right‐handed. Besides these general criteria, inclusion was based on performance in a previous behavioural session with the same ambiguous plaid stimuli. An average perceptual phase duration of >4 s and a balance of at least 80/20 between the two percepts in each possible stimulus configuration (pattern and component perception, see Section 2.2) were required to be selected for the fMRI session. The study was approved by the local ethics committee, and participants gave written informed consent.

### 2.2 Stimuli

**FIGURE 1:**
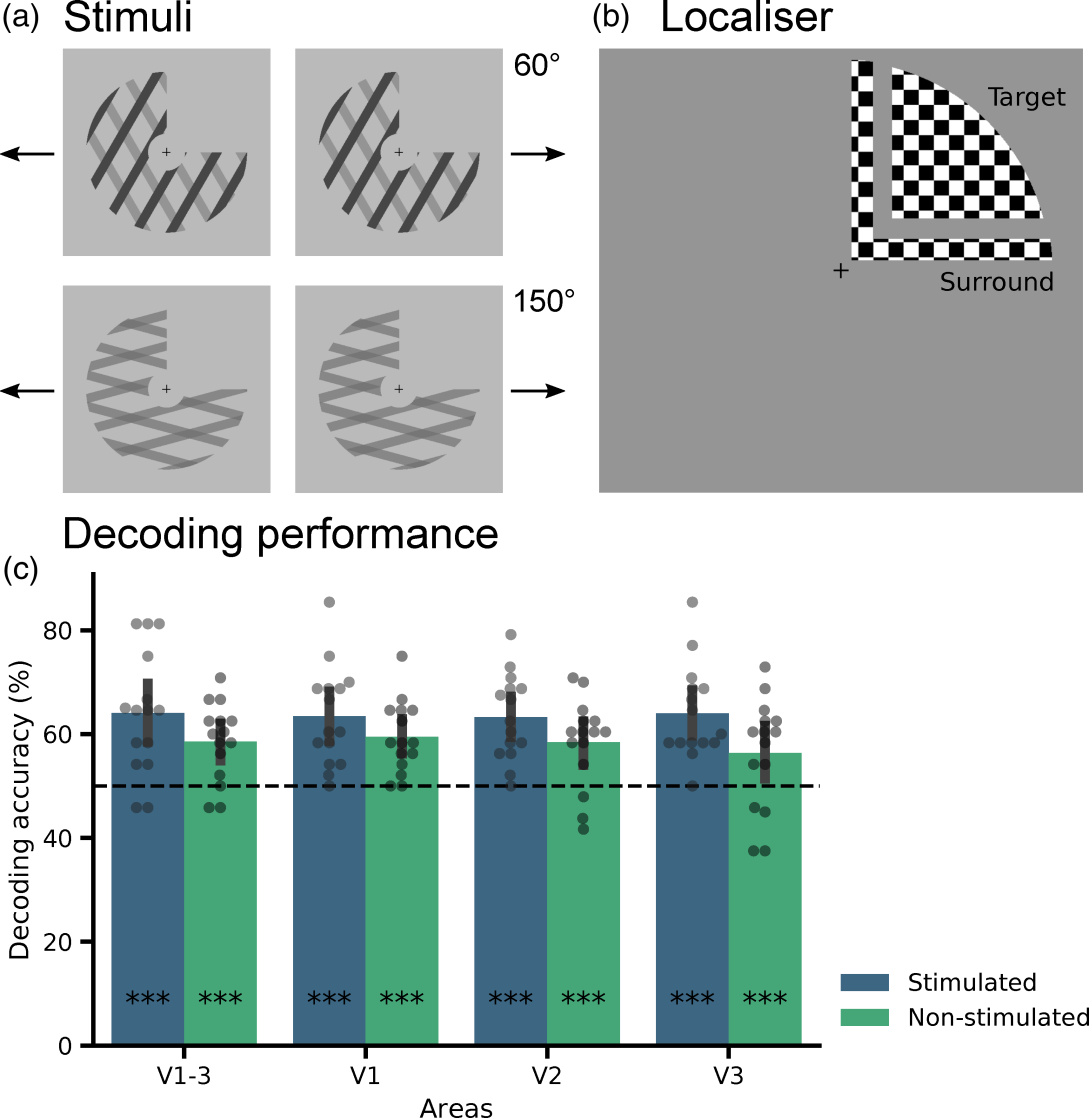
Stimuli and main results. **(**a) Ambiguous moving plaid stimuli were presented in four different stimulus configurations, which differed in the angle between the two component gratings (60° or 150°) and the overall motion direction of the resulting pattern (leftward or rightward). (b) The surround stimulus mapped the border between stimulated and non‐stimulated regions, and the target stimulus mapped the non‐stimulated quadrant (each presented in separate blocks, separated by fixation blocks). (c) Classifier accuracy discriminating component and pattern perception across all stimulus configurations for stimulated and non‐stimulated regions of early retinotopic areas. Error bars represent 95% confidence interval (CI). *p < .05, **p < .01, ***p < .001

Plaid stimuli were created by superimposing two individual component square‐wave gratings (van Kemenade, Seymour, Christophel, Rothkirch, & Sterzer, 2014). The stimuli were designed to be perceptually ambiguous, yielding bistable perception with spontaneous alternations between perception of either the two components moving in different directions (‘component perception’) or of one pattern moving in the average direction of the two gratings (‘pattern perception’). The angle between the components could be 60° or 150°, but for both angles the average motion direction between the two gratings was horizontal, either leftward or rightward, resulting in four stimulus configurations (60° left, 60° right, 150° left, 150° right) that all elicited bistability between component and pattern perception (see Figure 1a). fMRI results were pooled across these four stimulus configurations, as they were not relevant to the purpose of the present study. The individual gratings had a spatial frequency of 0.5 cycles per degree of visual angle and a duty cycle of 0.3. The term ‘duty cycle’ refers to the proportion of the width of the darker bars within one cycle of the grating. The speed of the individual gratings was 1.3 cycles/s for the 60° stimuli, and 0.39 cycles/s for the 150° stimuli. The speed of the resulting plaid stimuli was 1.5 cycles/s for all stimulus configurations. The plaid stimuli were presented within a centred annulus with a diameter of 13° of visual angle, and the upper right quadrant was occluded, that is, had the same luminance as the background (Figure 1a). In the centre of the annulus, which had a diameter of 3°, a fixation cross was presented. The background surrounding the stimuli had a luminance of 40 cd/m^2^. The luminance of the gratings of the 150° stimuli was 14 cd/m^2^. For the 60° stimuli, the two component gratings differed in luminance: one grating had 2 cd/m^2^, the other 20 cd/m^2^. The luminance of the intersections of the gratings was determined in pilot experiments that aimed at approximate equiprobability of component and pattern perception for all stimulus types and resulted in an intersection luminance of 9 cd/m^2^ for the 150° stimuli and 2 cd/m^2^ for the 60° stimuli.

### 2.3 Procedure

The stimuli were presented on a screen at the end of the MRI scanner bore. Participants laid in the scanner in supine position and viewed the stimuli on the screen through an angled mirror. They were asked to fixate on the central fixation cross and report their percept (pattern or component perception) by button presses. They had to report their percept as soon as the stimulus was presented, and press a button anytime their percept changed. A pattern percept was reported with the right index finger, and a component percept with the right middle finger. Each run comprised eight trials, lasting 60 s each, during which a plaid stimulus was continuously presented in one of the four stimulus configurations. Each trial was followed by a 10 s fixation interval, during which only the fixation cross was presented. Each stimulus configuration was presented twice per run in pseudorandomised order. There were six runs in total.

After the main experiment, two functional localisers were presented. The first was a stimulus localiser. Here, each stimulus from the main experiment was presented for 12 s, followed by fixation for 8 s, in a block‐design. Different from the main experiment, participants were asked to fixate only and not report their perception. All conditions were presented four times in total. This functional stimulus localiser allowed for selection of voxels that were activated by the stimuli used in the main experiment. Furthermore, we used a functional localiser that mapped the non‐stimulated region and was designed to preclude any spill‐over of activity from the stimulated region, similar to the localiser of Smith and Muckli (2010). During this localiser, participants viewed contrast‐reversing checkerboard stimuli (4 Hz), which were again presented for 12 s each, followed by 8 s of fixation. Each condition was repeated 8 times. The localiser contained ‘surround stimuli’, mapping the border between stimulated and non‐stimulated regions, and ‘target stimuli’, mapping the non‐stimulated region. The surround stimulus was presented at 0.5° of visual angle diagonally from the fixation cross, mapping the outer 1° of the non‐stimulated quadrant. The checkerboard representing the non‐stimulated quadrant, that is, the target stimulus, was presented at 1° diagonally from the surround stimulus (see Figure 1b). Thus, the target region, from which voxels were selected for our decoding analysis of the non‐stimulated quadrant, was ~2° away from the stimulated region. The scanning session ended with a structural T1 scan (MPRAGE). Standard phase‐encoded retinotopic mapping was performed in a separate scanning session to define regions V1‐3.

### 2.4 Scanning parameters

Functional MRI data were acquired using a 3 T TIM Trio scanner (Siemens, Erlangen, Germany), equipped with a 12‐channel head‐coil. A gradient echo EPI sequence was used (TR: 2 s, TE: 30 ms, flip angle: 80°, slice thickness: 2.3 mm, gap: 10%, voxel size 2.3 × 2.3 × 2.53 mm). A total of 280 volumes were acquired for each run of the main experiment, 163 volumes for the stimulus localiser, 163 volumes for the non‐stimulated quadrant localiser, 123 volumes per run (3 in total) for the polar angle retinotopic mapping, and 102 volumes per run (3 in total) for eccentricity mapping, each containing 29 slices oriented parallel to the calcarine sulcus and acquired in ascending order. Anatomical images were obtained using an MPRAGE sequence (TR: 1.9 s, TE: 2.52 ms, flip angle: 9°).

### 2.5 Eye movements

Eye movements were recorded with an iView Xtm MRI‐LR system [SensoMotoric Instruments (SMI), Teltow, Germany] using a sampling rate of 50 Hz. Due to technical difficulties, no usable eye tracking data were obtained for four participants, and for one run of a fifth participant. The eye tracking data were used in a control analysis to discard runs with poor fixation performance. To determine fixation performance, a radius of 1.5° from fixation was defined as the fixation area. Eye movements beyond this area were considered as outliers. Data were detrended and mean‐corrected to determine the number of these outliers, and runs in which eye movements extended beyond 1.5° of fixation in more than 5% of all data points were excluded. A total of 10 runs distributed across 5 participants were excluded in the control analysis based on eye tracking exclusion criteria.

### 2.6 fMRI analysis

The fMRI data were preprocessed and analysed using SPM12. First, the functional images were realigned to correct for head motion, after which they were coregistered with the structural image obtained in the same session. Then, both functional and structural images were coregistered with the structural image obtained in the retinotopy session. No normalisation or smoothing was applied, as is common for studies using multi‐voxel pattern analysis (MVPA).

A general linear model (GLM) was set up in which each regressor modelled all trials belonging to a given stimulus configuration and percept, resulting in eight regressors of interest. Motion parameters as well as a regressor modelling fixation in between trials were included as regressors of no interest. If participants reported only one percept for a certain condition, the other percept of that condition could not be modelled in that run; therefore, such runs were excluded. This affected all runs from one participant, and another seven runs distributed across three participants.

### 2.7 ROI definition

Regions of interest (ROIs) were defined with similar methods as those used by Smith and Muckli (2010). First, regions V1–V3 were defined using standard retinotopic mapping procedures. Within regions V1–V3, only the voxels that showed significant positive response to the stimulated region (t‐contrast stimulus > fixation, *p* < .01 uncorr.) in our stimulus localiser were selected. For the non‐stimulated region, the following procedure was used. First, we defined a region from the contrast non‐stimulated target area > surround (*p* < .01 uncorr). Then, in order to ensure that these voxels were not responsive to the stimulated region, we further selected from this region only the voxels that met these criteria: significant positive response to the non‐stimulated target area alone (*t* > 1.65, *p* < .01 uncorr.), no significant response to the stimulated area alone (*t* < 1.65, *p* > .01 uncorr.), and no significant response to the surround region (*t* < 1.65, *p* > .01 uncorr.).

The stimulated ROIs were naturally larger than the non‐stimulated ROIs, as the stimulus spanned three quadrants compared to one occluded quadrant. Furthermore, our strict criteria for selecting non‐stimulated voxels outlined above meant we only selected a small sample of the voxels corresponding to the occluded quadrant. To correct for potential biases induced by this difference in ROI size, we performed an additional control analysis with smaller stimulated ROIs that had the same number of voxels as their non‐stimulated counterpart ROI. These ROIs were generated by manually selecting voxels corresponding to the stimulus quadrant immediately opposite the occluded quadrant, in our case the quadrant in the upper left visual field. As such, we selected voxels in the right hemisphere below the calcarine sulcus. From these voxels, we randomly selected *n* voxels, with *n* being the number of voxels of the non‐stimulated ROI for that particular visual area (V1–V3) and participant. For two participants, not enough voxels were available in the respective stimulated quadrant of V1 to match the number of voxels from the non‐stimulated V1 ROI. For these two participants, we therefore used all the voxels available in the stimulated quadrant and thus had slightly less voxels in stimulated V1 ROI compared to the non‐stimulated V1 ROI (for one participant 12 stimulated voxels vs. 15 non‐stimulated voxels, for the other participant 6 stimulated voxels vs. 24 non‐stimulated voxels).

Data from a standard hMT+/V5 localiser were available for 10 of our subjects. Individual hMT+/V5 ROIs were defined by selecting voxels from the contrast moving dots > static dots (*p* < .001 uncorr.) whilst taking anatomical landmarks into account (Dumoulin, 2000).

### 2.8 MVPA

MVPA was performed using The Decoding Toolbox (Hebart, Görgen, & Haynes, 2015), which implements the LibSVM software (http://www.csie.ntu.edu.tw/wcjlin/libsv). A linear support vector machine was trained to discriminate pattern from component percepts based on the beta images resulting from the GLM. As the GLM already included grand mean scaling of the data, no additional scaling was performed. The classification was performed for each stimulus configuration separately. Classifier performance was tested using a leave‐one‐run‐out cross‐validation approach. Training was carried out on all but one run, which served as the test data. This was repeated until all runs had served as a test run once. The decoding accuracy was averaged across cross‐validations and then across conditions. Permutation testing was conducted to determine the significance at the group level as described by Stelzer, Chen, and Turner (2013). In brief, we provided the classifier with all possible combinations of shuffled label assignments for each participant and performed the decoding procedure for each label assignment. Then, we randomly selected one of these decoding accuracies from each participant and calculated the mean decoding accuracy. This procedure of random selection and calculation of mean decoding accuracy was repeated 10,000 to generate a distribution of decoding accuracies. We then used a cut‐off of 95% to determine significance of our results.

### 2.9 Univariate analysis

In order to further understand the neural mechanisms involved, we additionally performed a univariate analysis contrasting component with pattern percepts and vice versa. To this end, we used the same native‐space data used for our MVPA analysis, with the same GLM. We extracted the beta values for the contrasts patterns > baseline and components > baseline from the respective native‐space ROIs for each subject. We then performed repeated‐measures ANOVAs on these beta values with the factors Region (stimulated vs. non‐stimulated) and Percept (patterns vs. components). As in the multivariate approach, we first analysed the ROIs comprising V1–V3, and then analysed each region separately.

We performed the same analysis on our hMT+/V5 ROIs, where we expected to see more activity for components than patterns, as shown by previous studies (Castelo‐Branco et al., 2002; Grassi et al., 2018).

## 3 RESULTS

### 3.1 Phase durations

The mean perceptual phase duration of the 60° stimuli (averaged across leftward and rightward moving stimuli) was 7.4 s for components (*SD* = 8.6) and 9.9 s for patterns (*SD* = 4.6). For the 150° stimuli, mean phase duration for components was 8.2 s (*SD* = 7.5) and for patterns 4.9 s (*SD* = 1.7).

### 3.2 Decoding percepts

As displayed in Figure 1c, significant above‐chance decoding performance was obtained for both stimulated (64.1%, *p* < .001) and non‐stimulated (58.6%, *p* < .001) regions of areas V1–V3 together. Decoding performance also reached significance in each of the retinotopic areas separately (V1: 63.4% stimulated, 59.4% non‐stimulated; V2: 63.3% stimulated, 58.4% non‐stimulated; V3: 64% stimulated, 56.3% non‐stimulated; all *p* < .001).

### 3.3 Control analysis discarding runs with poor fixation performance

**FIGURE 2:**
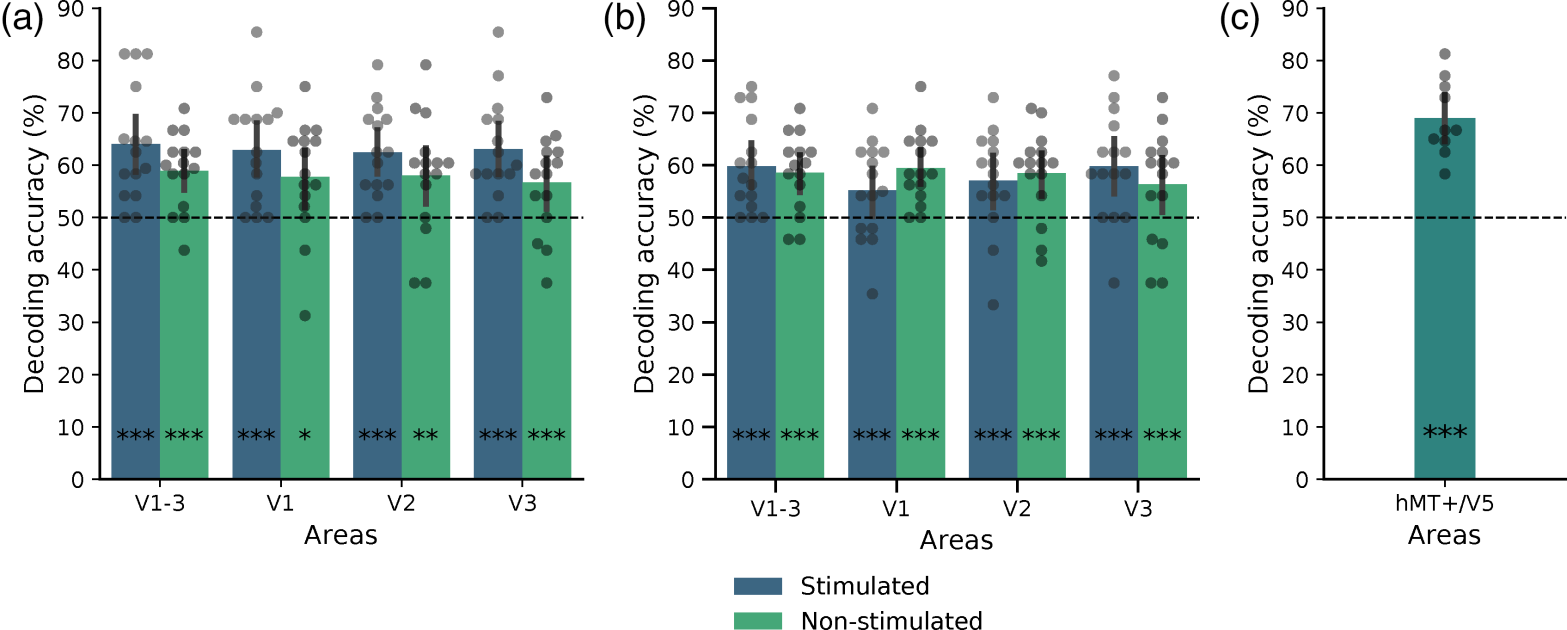
Results of control analyses**. (**a and b) Classifier accuracy discriminating component and pattern perception across all stimulus configurations for stimulated and non‐stimulated regions of early retinotopic areas. Error bars represent 95% confidence interval (CI). *p < .05, **p < .01, ***p < .001. (a) In this analysis, runs with poor fixation performance were excluded. (b) In this analysis, the number of voxels in stimulated V1 ROIs matched those of non‐stimulated V1 ROIs. (c) Classifier accuracy discriminating component and pattern perception across all stimulus configurations for area hMT+/V5

Overall fixation accuracy across all participants was 97.3%. Despite this high accuracy, we performed a control analysis discarding runs with fixations more than 5% outside of our fixation ROI. As displayed in Figure 2a, significant above‐chance decoding performance was obtained for both stimulated (64.0%, *p* < .001) and non‐stimulated (58.9%, *p* < .001) regions of areas V1–V3 together. Decoding performance also reached significance in each of the retinotopic areas separately (V1: 62.9% stimulated, *p* < .001, 57.8% non‐stimulated, *p* = .015; V2: 62.4% stimulated, *p* < .001, 58.0% non‐stimulated, *p* = .007; V3: 63.0% stimulated, *p* < .001, 56.7% non‐stimulated, *p* < .001).

### 3.4 Control analysis correcting for the difference in number of voxels between stimulated and non‐stimulated ROIs

In this analysis, we decoded from stimulated and non‐stimulated ROIs that were matched in size. As displayed in Figure 2b, significant above‐chance decoding performance was obtained for both stimulated (60.9%, *p* < .001) and non‐stimulated (58.6%, *p* < .001) regions of areas V1–V3 together. Decoding performance also reached significance in each of the retinotopic areas separately (V1: 55.2% stimulated, 59.4% non‐stimulated; V2: 56.5% stimulated, 58.4% non‐stimulated; V3: 59.2% stimulated, 56.3% non‐stimulated, all *p* < .001).

### 3.5 Control analysis decoding from hMT+/V5

This proof‐of‐concept analysis revealed that component and pattern percepts could be decoded from hMT+/V5 with high accuracy (69.0%, *p* < .001, see Figure 2c).

### 3.6 Univariate analysis

An ANOVA with the factors Region (stimulated vs. non‐stimulated) and Percept (patterns vs. components) on the large ROIs (V1–V3) showed a significant main effect of Region [*F*(1,14) = 52.54, *p* < .001, *n*_*p*_^2^ (partial eta squared) = 0.79], as well as a significant main effect of Percept *F*(1,14) = 5.16, *p* = .039, *n*_p_^2^ = 0.27). Furthermore, there was a significant Region x Percept interaction [*F*(1,14) = 10.25, *p* = .006, *n*_*p*_^2^ = 0.42]. Paired‐samples *t*‐tests showed that the interaction was driven by significantly higher activation for patterns (M = −14.24, *SD* = 21.14) compared to components (M = −22.33, *SD* = 21.70) in the non‐stimulated regions (*t*[14] = 3.10, *p* = .008), whereas no significant difference was found between patterns (M = 43.47, *SD* = 15.80) and components (M = 40.68, *SD* = 16.90) in stimulated regions (*t*[14] = 1.12, *p* = .280). This pattern was generally also present in each visual area separately, with significant main effects of Region in all areas [V1: *F*(1,14) = 42.98, *p* < .001, *n*_*p*_^2^ = 0.75; V2: *F*(1,14) = 58.20, *p* < .001, *n*_*p*_^2^ = 0.81; V3: *F*(1,14) = 75.49, *p* < .001, *n*_*p*_^2^ = 0.84]. The main effect of Percept reached significance only in V1 [*F*(1,14) = 4.70, *p* = .048, *n*_*p*_^2^ = 0.25], but showed effects in the same direction in V2 [*F*(1,14) = 4.32, *p* = .057, *n*_*p*_^2^ = 0.24] and V3 [*F*(1,14) = 3.88, *p* = .069, *n*_*p*_^2^ = 0.22]. The Region x Percept interaction was significant in both V2 (*F*(1,14) = 10.87, *p* = .005, *n*_*p*_^2^ = 0.44) and V3 [*F*(1,14) = 12.00, *p* = .004, *n*_*p*_^2^ = 0.46], and was driven in both areas by a significantly higher activation for patterns (V2: M = −13.80, *SD* = 19.40; V3: M = −8.32, *SD* = 14.69) compared to components (V2: M = −21.95, *SD* = 18.93; V3: M = −15.73, *SD* = 13.23) in non‐stimulated regions [V2: *t*(14) = 2.79, *p* = .014; V3: *t*(14) = 3.18, *p* = .007]. The difference between patterns (V2: M = 41.20, *SD* = 14.92; V3: M = 41.26, *SD* = 15.44) and components (V2: M = 38.24, *SD* = 15.86; V3: M = 40.02, *SD* = 16.00) was not significant in stimulated regions [V2: *t*(14) = 1.18, *p* = .283; V3: *t*(14) = 0.52, *p* = .614]. Area V1 showed a similar but non‐significant interaction in the same direction [*F*(1,14) = 4.06, *p* = .064, *n*_*p*_^2^ = 0.23].

**FIGURE 3:**
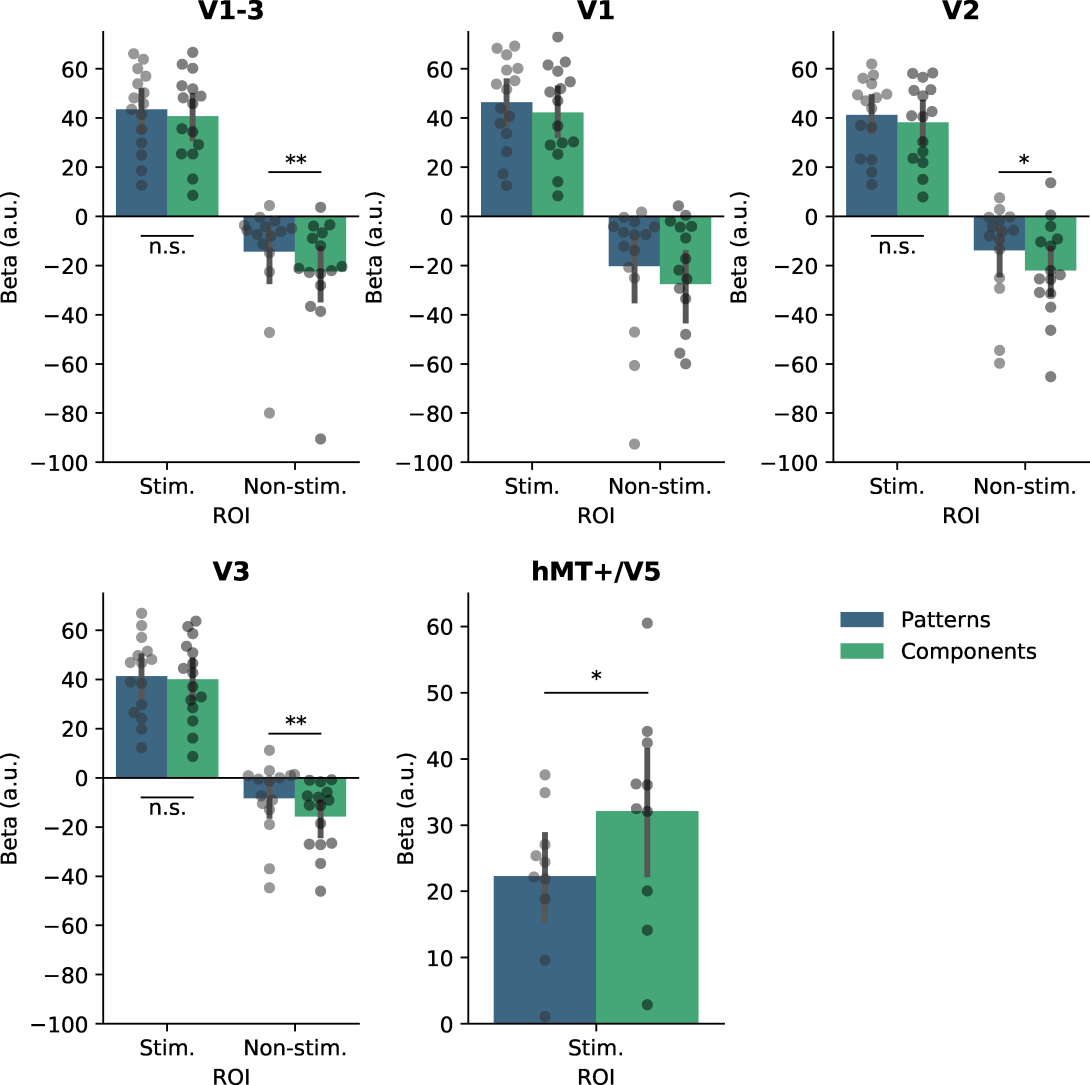
Results of univariate analysis. Beta values are displayed for patterns and components in each ROI. Early visual areas generally showed increased activity for patterns compared to components in non‐stimulated areas. In contrast, we observed more activity for components than patterns in area hMT+/V5. Significance labels are added for post‐hoc *t*‐tests (*p < .05, **p < .01, ***p < .001, n.s. = not significant). Since the Region x Percept interaction did not reach significance in V1, no post‐hoc *t*‐tests were performed for this region, but the results point in the same direction as the other early visual regions. Error bars represent 95% confidence interval (CI)

Our ROI analysis on area hMT+/V5 showed significantly more activity for components than patterns [*t*(9) = −2.33, *p* = .045, see Figure 3].

## 4 DISCUSSION

Our findings show that the current perceptual state during bistability can be decoded from fMRI signal patterns not only in stimulated early visual regions, which is in line with previous studies (Haynes & Rees, 2005), but crucially also in non‐stimulated retinotopic visual cortex, which did not receive any bottom‐up input. This suggests that non‐stimulated regions of early visual cortex contain information not only about visual stimulation in the surrounding context, as previously shown (Smith & Muckli, 2010), but even about conscious perception independent of visual stimulation *per se*. This is in line with current theories that model bistable perception within the framework of predictive processing (Brascamp, Sterzer, Blake, & Knapen, 2018; Hohwy et al., 2008). According to this view, ambiguous stimuli (such as the bistable moving plaids used here) provide equally strong sensory evidence for two different percepts, but the currently dominant percept establishes an implicit prediction regarding the cause of the sensory input. This prediction is thought to stabilize the current perceptual state through feedback from higher to lower hierarchical levels, while sensory evidence for the currently suppressed perceptual interpretation elicits prediction errors that act to destabilize the current percept, eventually leading to a perceptual change (Weilnhammer et al., 2021; Weilnhammer, Stuke, Hesselmann, Sterzer, & Schmack, 2017). Here, we provide evidence supporting the notion of feedback signalling of predictions in bistable perception.

There have been other studies that showed neural activity in visual areas that were not directly stimulated. These include studies on object perception (Williams et al., 2008), feature‐based attention (Serences & Boynton, 2007), visual scene perception (Smith & Muckli, 2010), and illusions like the Kanizsa triangle (Kok, Bains, van Mourik, Norris, & de Lange, 2016), apparent motion (Chong, Familiar, & Shim, 2016; Muckli, Kohler, Kriegeskorte, & Singer, 2005), or the bistable Gestalt illusion (Grassi, Zaretskaya, & Bartels, 2017 **)**. Our study is in line with this earlier work, which underlines the idea that long‐range connections carry feedback signals from higher areas back to early visual cortex. However, it is distinct from these findings in the key aspect that it shows that such feedback signals in non‐stimulated visual areas carry information about the *subjective interpretation* of an ambiguous stimulus, where the physical properties of the stimulus are stable, while the conscious perception of the participant alternates between two alternative interpretations. Bistable motion quartets inducing apparent motion also show activity along the non‐stimulated motion path depending on conscious interpretation, but this activity underlies the reconstruction of an illusory percept, that is, of a stimulus that is not actually there. In our study, the activity reflected feedback signals about a stimulus that was always physically present, but was interpreted in different ways over time. As such, our results do not only support the general idea that predictions are sent back to early visual cortex, but importantly that they are involved in the subjective interpretation of an ambiguous stimulus.

Our univariate results showed significantly more activation for patterns than components in non‐stimulated early visual areas. Increased activation for patterns in early visual cortex has been reported in previous studies as well (Grassi et al., 2018; Wilbertz, Ketkar, Guggenmos, & Sterzer, 2018). We observed this pattern only in non‐stimulated areas, which resembles the results by Grassi et al. (2017) that a global Gestalt percept induced more activity in the illusory percept regions in early visual cortex than a local Gestalt percept. The fact that we observed this effect in non‐stimulated regions only seems to support the hypothesis that it is driven by feedback mechanisms, as indicated by findings from Kok et al. (2016) who found enhanced activity for illusory percepts only in deep cortical layers that process feedback signals. As such, our univariate results support our multivariate results. Since it has been shown that attentional mechanisms can also drive perceptual effects in non‐stimulated areas (Serences & Boynton, 2007), it is possible that attention to the current percept might have contributed to the results. However, since we found opposite univariate patterns in early visual cortex (more activity for pattern percepts) and area hMT+/V5 (more activity for component percepts), feedback mechanisms seem a more likely explanation. On a similar note, it has been reported that people blink more during pattern perception compared to component perception (Brych, Murali, & Händel, 2021), which could be an alternative explanation for the increased BOLD response in visual cortex (Hupé et al., 2012). However, again the opposite pattern in early visual cortex versus hMT+/V5 seems to rather point at the involvement of feedback mechanisms.

We suggest that the percept‐related information that we found in non‐stimulated regions of early visual areas most likely arises from feedback signalling that originates from higher‐level areas concerned with the computation of component vs. pattern motion perception, such as area hMT+/V5 (Castelo‐Branco et al., 2002; Duarte, Costa, Martins, & Castelo‐Branco, 2017; Grassi et al., 2018). Research on bistable plaid motion has shown that hMT+/V5 is concerned with the disambiguation of bistable plaids into pattern and component motion (Castelo‐Branco et al., 2002), and that it sends information back to early visual cortex during this process (Duarte et al., 2017). Furthermore, effective connectivity analyses have shown that apparent motion induced activation of non‐stimulated visual regions along the illusory apparent motion path is associated with enhanced feedback signalling from area hMT+/V5 (Sterzer, Haynes, & Rees, 2006), which has been shown to be causally involved in such apparent motion perception in a later TMS study (Vetter, Grosbras, & Muckli, 2015). Considering these studies, it seems plausible that area hMT+/V5 is also involved in predictive feedback signalling to non‐stimulated areas during bistable plaid motion perception, and that our results thus reflect predictive feedback signalling coming from this area. Our significant decoding results in hMT+/V5 support the idea that this area generates the predictions that are sent back to early visual areas during bistable perception, though future studies will have to provide direct causal evidence. There are other potential origins of feedback signalling in bistable plaid perception, as several studies have shown involvement of frontoparietal areas in bistable perception (Brascamp et al., 2018; Grassi et al., 2018; Weilnhammer et al., 2021). Recent evidence suggests that hMT+/V5 might signal perceptual conflict to and receive signals from frontal areas to resolve this conflict, making hMT+/V5 a hub for receiving and relaying feedback signals from and to frontal cortex (Weilnhammer et al., 2021). As our study was focused on visual cortex, we were unable to verify the involvement of areas outside visual cortex. However, our results support the idea of hMT+/V5 as a source of feedback signals to early visual cortex in bistable perception.

In conclusion, our current results provide compelling support for the notion that conscious perception reflects an internal model that generates predictions about the current state of the world, and that these predictions are fed back to the lowest levels of sensory processing to enable inferences regarding the sensory input.

## CONFLICT OF INTEREST

The authors declare no conflict of interest.

## AUTHOR CONTRIBUTIONS

**Bianca M. van Kemenade**: Conceptualization, methodology, software, formal analysis, writing—original draft and writing—review & editing. **Philipp Sterzer**: Conceptualization, methodology, writing—review & editing, supervision, and funding acquisition. **Gregor Wilbertz:** Software, investigation, writing—review & editing. **Annalena Müller**: Investigation, formal analysis, writing—review & editing.

## Data Availability

The data that support the findings of this study are available on request from the corresponding author. The data are not publicly available due to privacy or ethical restrictions.

## References

[hbm25731-bib-0001] Brascamp, J. , Sterzer, P. , Blake, R. , & Knapen, T. (2018). Multistable perception and the role of the frontoparietal cortex in perceptual inference. Annual Review of Psychology, 69, 77–103. 10.1146/annurev-psych-010417-085944 28854000

[hbm25731-bib-0002] Brych, M. , Murali, S. , & Händel, B. (2021). The role of blinks, microsaccades and their retinal consequences in bistable motion perception. Frontiers in Psychology, 12, 647256. 10.3389/fpsyg.2021.647256 33897552 PMC8061730

[hbm25731-bib-0003] Castelo‐Branco, M. , Formisano, E. , Backes, W. , Zanella, F. , Neuenschwander, S. , Singer, W. , & Goebel, R. (2002). Activity patterns in human motion‐sensitive areas depend on the interpretation of global motion. Proceedings of the National Academy of Sciences, 99(21), 13914–13919. 10.1073/pnas.202049999 PMC129797 12368476

[hbm25731-bib-0004] Chong, E. , Familiar, A. M. , & Shim, W. M. (2016). Reconstructing representations of dynamic visual objects in early visual cortex. Proceedings of the National Academy of Sciences of the United States of America, 113(5), 1453–1458. 10.1073/pnas.1512144113 26712004 PMC4747748

[hbm25731-bib-0005] De Lange, F. P. , Heilbron, M. , & Kok, P. (2018). How do expectations shape perception? Trends in Cognitive Sciences, (June). 22(9), 764–779. 10.1016/j.tics.2018.06.002 30122170

[hbm25731-bib-0006] Duarte, J. V. , Costa, G. N. , Martins, R. , & Castelo‐Branco, M. (2017). Pivotal role of hMT+ in long‐range disambiguation of interhemispheric bistable surface motion. Human Brain Mapping, 38(10), 4882–4897. 10.1002/hbm.23701 28660667 PMC6866998

[hbm25731-bib-0007] Dumoulin, S. O. (2000). A New Anatomical Landmark for Reliable Identification of Human Area V5/MT: a Quantitative Analysis of Sulcal Patterning. Cerebral Cortex, 10(5), 454–463. 10.1093/cercor/10.5.454 10847595

[hbm25731-bib-0008] Friston, K. (2005). A theory of cortical responses. Philosophical Transactions of the Royal Society B: Biological Sciences, 360, 815–836. 10.1098/rstb.2005.1622 PMC1569488 15937014

[hbm25731-bib-0009] Grassi, P. R. , Zaretskaya, N. , & Bartels, A. (2017). Scene segmentation in early visual cortex during suppression of ventral stream regions. NeuroImage, 146, 71–80. 27847346 10.1016/j.neuroimage.2016.11.024

[hbm25731-bib-0010] Grassi, P. R. , Zaretskaya, N. , & Bartels, A. (2018). A Generic Mechanism for Perceptual Organization in the Parietal Cortex. Journal of Neuroscience, 38(32), 7158–7169. 30006362 10.1523/JNEUROSCI.0436-18.2018 PMC6596091

[hbm25731-bib-0011] Haynes, J. D. , & Rees, G. (2005). Predicting the stream of consciousness from activity in human visual cortex. Current Biology, 15(14), 1301–1307. 10.1016/j.cub.2005.06.026 16051174

[hbm25731-bib-0012] Hebart, M. N. , Görgen, K. , & Haynes, J.‐D. (2015). The Decoding Toolbox (TDT): a versatile software package for multivariate analyses of functional imaging data. Frontiers in Neuroinformatics, 8(January), 1–18. 10.3389/fninf.2014.00088 PMC4285115 25610393

[hbm25731-bib-0013] Hohwy, J. , Roepstorff, A. , & Friston, K. (2008). Predictive coding explains binocular rivalry: An epistemological review. Cognition, 108(3), 687–701. 10.1016/j.cognition.2008.05.010 18649876

[hbm25731-bib-0029] Hupé, J.‐M. , Bordier, C. , & Dojat, M. (2012). A BOLD signature of eyeblinks in the visual cortex. Neuroimage, 61(1), 149–161. 10.1016/j.neuroimage.2012.03.001 22426351

[hbm25731-bib-0014] Kok, P. , Bains, L. J. , van Mourik, T. , Norris, D. G. , & de Lange, F. P. (2016). Selective activation of the deep layers of the human primary visual cortex by top‐down feedback. Current Biology, 26(3), 371–376. 10.1016/j.cub.2015.12.038 26832438

[hbm25731-bib-0015] Muckli, L. , De Martino, F. , Vizioli, L. , Petro, L. S. , Smith, F. W. , Ugurbil, K. , … Yacoub, E. (2015). Contextual feedback to superficial layers of V1. Current Biology, 25(20), 2690–2695. 10.1016/j.cub.2015.08.057 26441356 PMC4612466

[hbm25731-bib-0016] Muckli, L. , Kohler, A. , Kriegeskorte, N. , & Singer, W. (2005). Primary visual cortex activity along the apparent‐motion trace reflects illusory perception. PLoS Biology, 3(8), e265. 10.1371/journal.pbio.0030265 16018720 PMC1175820

[hbm25731-bib-0017] Rao, R. P. N. , & Ballard, D. H. (1999). Predictive coding in the visual cortex: a functional interpretation of some extra‐classical receptive‐field effects. Nature Neuroscience, 2(1), 79–87. 10.1038/4580 10195184

[hbm25731-bib-0018] Revina, Y. , Petro, L. S. , & Muckli, L. (2017). Cortical feedback signals generalise across different spatial frequencies of feedforward inputs. NeuroImage, 180, 280–290. 10.1016/j.neuroimage.2017.09.047 28951158

[hbm25731-bib-0019] Serences, J. T. , & Boynton, G. M. (2007). Feature‐Based Attentional Modulations in the Absence of Direct Visual Stimulation. Neuron, 55(2), 301–312. 10.1016/j.neuron.2007.06.015 17640530

[hbm25731-bib-0020] Smith, F. W. , & Muckli, L. (2010). Nonstimulated early visual areas carry information about surrounding context. Proceedings of the National Academy of Sciences, 107(46), 20099–20103. 10.1073/pnas.1000233107 PMC2993348 21041652

[hbm25731-bib-0021] Stelzer, J. , Chen, Y. , & Turner, R. (2013). Statistical inference and multiple testing correction in classification‐based multi‐voxel pattern analysis (MVPA): Random permutations and cluster size control. NeuroImage, 65, 69–82. 10.1016/j.neuroimage.2012.09.063 23041526

[hbm25731-bib-0022] Sterzer, P. , Haynes, J. D. , & Rees, G. (2006). Primary visual cortex activation on the path of apparent motion is mediated by feedback from hMT+/V5. NeuroImage, 32(3), 1308–1316. 10.1016/j.neuroimage.2006.05.029 16822682

[hbm25731-bib-0023] van Kemenade, B. M. , Seymour, K. , Christophel, T. B. , Rothkirch, M. , & Sterzer, P. (2014). Decoding pattern motion information in V1. Cortex, 57, 177–187. 10.1016/j.cortex.2014.04.014 24905972

[hbm25731-bib-0024] Vetter, P. , Grosbras, M. H. , & Muckli, L. (2015). TMS over V5 disrupts motion prediction. Cerebral Cortex, 25(4), 1052–1059. 10.1093/cercor/bht297 24152544 PMC4380002

[hbm25731-bib-0025] Weilnhammer, V. , Fritsch, M. , Chikermane, M. , Eckert, A. , Kanthak, K. , Stuke, H. , … Sterzer, P. (2021). An active role of inferior frontal cortex in conscious experience. Current Biology, 31, 2868–2880. 33989530 10.1016/j.cub.2021.04.043

[hbm25731-bib-0026] Weilnhammer, V. , Stuke, H. , Hesselmann, G. , Sterzer, P. , & Schmack, K. (2017). A predictive coding account of bistable perception ‐ a model‐based fMRI study. PLoS Computational Biology, 13(5), 1–21. 10.1371/journal.pcbi.1005536 PMC5448813 28505152

[hbm25731-bib-0027] Wilbertz, G. , Ketkar, M. , Guggenmos, M. , & Sterzer, P. (2018). Combined fMRI‐ and eye movement‐based decoding of bistable plaid motion perception. NeuroImage, 171, 190–198. 29294388 10.1016/j.neuroimage.2017.12.094

[hbm25731-bib-0028] Williams, M. A. , Baker, C. I. , Op De Beeck, H. P. , Mok Shim, W. , Dang, S. , Triantafyllou, C. , & Kanwisher, N. (2008). Feedback of visual object information to foveal retinotopic cortex. Nature Neuroscience, 11(12), 1439–1445. 10.1038/nn.2218 18978780 PMC2789292

